# Lactate and Choline Metabolites Detected *In Vitro* by Nuclear Magnetic Resonance Spectroscopy Are Potential Metabolic Biomarkers for PI3K Inhibition in Pediatric Glioblastoma

**DOI:** 10.1371/journal.pone.0103835

**Published:** 2014-08-01

**Authors:** Nada M. S. Al-Saffar, Lynley V. Marshall, L. Elizabeth Jackson, Geetha Balarajah, Thomas R. Eykyn, Alice Agliano, Paul A. Clarke, Chris Jones, Paul Workman, Andrew D. J. Pearson, Martin O. Leach

**Affiliations:** 1 Cancer Research UK and EPSRC Cancer Imaging Centre, Division of Radiotherapy and Imaging, The Institute of Cancer Research and The Royal Marsden NHS Foundation Trust, London, United Kingdom; 2 Division of Molecular Pathology, The Institute of Cancer Research and The Royal Marsden NHS Foundation Trust, London, United Kingdom; 3 Division of Cancer Therapeutics, The Institute of Cancer Research and The Royal Marsden NHS Foundation Trust, London, United Kingdom; 4 Division of Clinical Studies. The Institute of Cancer Research and The Royal Marsden NHS Foundation Trust, London, United Kingdom; 5 Division of Imaging Sciences and Biomedical Engineering, King’s College London, St Thomas’ Hospital, London, United Kingdom; 6 Cancer Research UK Cancer Therapeutics Unit, The Institute of Cancer Research, London, United Kingdom; Spanish National Cancer Center, Spain

## Abstract

The phosphoinositide 3-kinase (PI3K) pathway is believed to be of key importance in pediatric glioblastoma. Novel inhibitors of the PI3K pathway are being developed and are entering clinical trials. Our aim is to identify potential non-invasive biomarkers of PI3K signaling pathway inhibition in pediatric glioblastoma using *in vitro* nuclear magnetic resonance (NMR) spectroscopy, to aid identification of target inhibition and therapeutic response in early phase clinical trials of PI3K inhibitors in childhood cancer. Treatment of SF188 and KNS42 human pediatric glioblastoma cell lines with the dual pan-Class I PI3K/mTOR inhibitor PI-103, inhibited the PI3K signaling pathway and resulted in a decrease in phosphocholine (PC), total choline (tCho) and lactate levels (p<0.02) as detected by phosphorus (^31^P)- and proton (^1^H)-NMR. Similar changes were also detected using the pan–Class I PI3K inhibitor GDC-0941 which lacks significant mTOR activity and is entering Phase II clinical trials. In contrast, the DNA damaging agent temozolomide (TMZ), which is used as current frontline therapy in the treatment of glioblastoma postoperatively (in combination with radiotherapy), increased PC, glycerophosphocholine (GPC) and tCho levels (p<0.04). PI-103-induced NMR changes were associated with alterations in protein expression levels of regulatory enzymes involved in glucose and choline metabolism including GLUT1, HK2, LDHA and CHKA. Our results show that by using NMR we can detect distinct biomarkers following PI3K pathway inhibition compared to treatment with the DNA-damaging anti-cancer agent TMZ. This is the first study reporting that lactate and choline metabolites are potential non-invasive biomarkers for monitoring response to PI3K pathway inhibitors in pediatric glioblastoma.

## Introduction

Approximately 40% of all pediatric brain tumors are astrocytomas (gliomas), and of these some 15–20% are malignant gliomas, i.e. high-grade (WHO grade III and IV) tumors [1], [2]. High-grade gliomas (HGGs) are very aggressive tumors and are one of the leading causes of cancer-related deaths in children with a median survival of just 12–15 months for children with glioblastoma [1], [3]. Although these tumors are morphologically similar to malignant gliomas that arise in adults, the molecular pathways of gliomagenesis in children differ substantially from those in adults, resulting in tumors that may arise at differing incidences in different anatomical sites compared to adults and which have a distinct underlying biology [3]–[8].

As well as numerous qualitative and quantitative differences in DNA copy number abnormalities between pediatric and adult HGG [3], childhood tumors are defined in part by the presence of specific somatic mutations in the gene encoding the histone H3.3 variant, *H3F3A* [7]. These mutations result in amino acid substitutions at two critical positions within the histone tail: the K27M mutation results in a lysine to methionine substitution and the G34R or G34V mutations result in glycine to arginine or valine substitutions. The K27 mutations are typically seen in younger pediatric patients with tumors arising in central locations e.g. brainstem or thalamus, whereas the G34 mutations arise in older pediatric/adolescent patients with tumors arising in the supratentorial (typically cerebral hemispheric) locations [3], [7], [8]. These histone H3 mutations are not seen in adult HGG beyond approximately 30 years of age and are mutually exclusive with the high frequency of *IDH1* mutations seen in adult populations between 35–45 years of age.

Despite these substantial molecular differences, both adult and childhood malignant gliomas are generally treated similarly with a combination of surgery, irradiation and alkylator-based chemotherapy, using agents such as temozolomide (TMZ), with the classic drug treatment being the Stupp regimen of postoperative radiotherapy with concomitant and adjuvant TMZ [9]. Even with the best protocols, these current treatment strategies provide dismal cure rates for pediatric glioblastoma patients, with TMZ adding only modest survival benefit at best [10], [11]. Therefore, research is continuing to unravel the key molecules and signaling pathways responsible for the oncogenesis of different childhood brain tumors with the aim that a new era of molecular based therapies will deliver major benefits for pediatric gliomas [1].

There is mounting evidence that the PI3K/AKT/mTOR signaling pathway is activated in pediatric glioblastoma and contributes to resistance to TMZ [12], [13], thus providing key targets for the treatment of pediatric glioblastoma. Numerous small-molecule inhibitors of the PI3K signaling pathway are being developed [14]–[16] and are progressing through Phase I/II clinical trials in adults with solid tumors, including glioma, and we are currently planning a first-in-child pediatric Phase I trial with an expansion cohort in pediatric glioblastoma.

For the clinical development and evaluation of new molecularly targeted therapies that inhibit signaling pathways, new methods for the assessment of changes in biological properties are required [17]. Non-invasive methods are of particular clinical importance in the study of childhood brain tumors, as they may avoid the need for (repeated) biopsy whilst still providing pharmacodynamic evidence of target or pathway inhibition. A recent study demonstrated the feasibility of [^18^F]FDG PET to monitor response to PI3K inhibition in adult patients with advanced solid tumors [18]. However, radiation exposure is a potential limitation of PET radioisotopes, particularly in children [19].

Magnetic resonance spectroscopy (MRS) offers the opportunity to investigate metabolic components of cells and tissues in physiological environments, non-invasively and without the use of radioactive reagents. The data are represented by a spectrum, in which the peaks correspond to different metabolites wherein peak areas can be measured and metabolite concentrations quantified [20]. MRS is a powerful tool for the assessment of brain tumors including pre-surgical diagnosis of tumor type and grade, monitoring of treatment response, and evaluation of tumor recurrence [21]–[23]. There are a number of metabolites that can be identified by standard brain proton (^1^H)-MRS, but only a few of them have a clinical significance in gliomas including N-acetylaspartate, choline-containing metabolites, creatine, myo-inositol, lactate, and lipids [21]. Our goal is to define the utility of *in vitro* nuclear magnetic resonance (NMR) and subsequently *in vivo* MRS in providing non-invasive pharmacodynamic biomarkers for the inhibition of the PI3K signaling pathway and to aid identification of target inhibition and therapeutic response in early phase clinical trials of PI3K pathway inhibitors in children with glioblastoma.

Using NMR, we have previously reported altered choline metabolism in response to inhibition of the PI3K signaling pathway with LY294002, wortmannin and the selective dual pan-Class I PI3K/mTOR inhibitor PI-103 in adult human cancer cell models [24], [25]. In this work, we have utilized *in vitro* ^1^H- and phosphorus (^31^P)-NMR to monitor metabolic changes following PI3K pathway inhibition by PI-103 and pan–Class I PI3K inhibitor GDC-0941 which lacks significant mTOR activity and is entering Phase II clinical trials, using the pediatric glioblastoma cell lines SF188 and KNS42. Metabolic changes were compared to biomarkers of treatment with the standard of care cytotoxic drug TMZ. We have also investigated potential mechanisms underlying the observed metabolic changes. We report distinct metabolic changes including a decrease in the levels of lactate, phosphocholine (PC) and total choline (tCho) following PI3K pathway inhibition with PI-103 or GDC-0941, whereas there was an increase in PC, glycerophosphocholine (GPC) and tCho following treatment with TMZ. Furthermore, the decrease in PC levels was associated with a decrease in the protein levels of choline kinase alpha (CHKA), the enzyme responsible for choline phosphorylation to form PC. A decrease in the protein expression levels of the facilitative glucose transporter (GLUT1) and the glycolytic enzymes hexokinase II (HK2) and lactate dehydrogenase alpha (LDHA) as well as a decrease in glucose uptake were also observed following PI3K pathway inhibition, suggesting reduced glycolytic flux as a mechanism for depletion of lactate.

## Materials and Methods

### Cell culture and treatment

The human pediatric glioblastoma (WHO grade IV) cell lines SF188 (a kind gift from Dr. Daphne Haas-Kogan, University of California San Francisco, San Francisco, CA, USA [26]) and KNS42 (obtained from Japan Cancer Research Resources cell bank [27]), have been extensively characterized previously [28]. Both cell lines are wildtype for *PTEN* and *PIK3CA* [13], [28] (data not shown). KNS42 is histone H3.3 (H3F3A) G34V mutant and SF188 is wildtype [4]. Cells were grown as monolayers in DMEM/F12 Ham’s medium+10% FCS in 5% CO2. Cell viability was routinely >90%, as judged by trypan blue exclusion. Both cell lines routinely tested negative for mycoplasma by PCR.

The two cell lines were treated with the dual pan-Class I PI3K/mTOR inhibitor PI-103 [14]–[16] (Sigma) and the pan-Class I PI3K inhibitor GDC-0941 [14]–[16] (Genentech) which lacks significant mTOR activity. SF188 cells were also treated with the cytotoxic drug TMZ (Sigma). GI_50_ values (concentrations causing 50% inhibition of proliferation of tumor cells) were determined using the MTS assay [29] following continuous exposure to compounds for 3 doubling times. Preliminary experiments were performed on both pediatric cell lines to measure levels of PC per cell at different cell number. This was to establish the cell number to be used for setting up the experiments such that the availability of PC per cell is similar in control and treated cells over the time course of treatment. The final time point was based on the length of the doubling time for the cell lines used (SF188 doubling time 26 hours and KNS42 doubling time 48 hours) and intermediate time points were selected to assess early detection of biomarkers. At the required time points, cells underwent trypsinization and trypan blue exclusion assay [30]. The effect of treatment on cell number was monitored by counting the number of viable attached cells in a treated flask and comparing that number with the number of attached cells in a control flask.

### Flow cytometry

Cell cycle analysis was performed as previously described [30]. Control and treated cells were harvested by trypsinization, washed in PBS and fixed in 70% ethanol. Fixed cells were washed and resuspended in PBS supplemented with 10 mg/ml RNase A (Sigma) and 40 mg/mL propidium iodide (Sigma). After 30 minutes of incubation at 37°C, cells were analyzed using BD LSRII flow cytometer (BD, San Jose, CA, USA). The cytometry data were analyzed using the WinMdi and Cylchred software (University of Wales College of Medicine, Cardiff, UK).

### Immunoblotting

Western blotting was performed as previously described [25]. Cells were lysed in lysis buffer (Cell Signaling) supplemented with a complete mini protease inhibitor cocktail (Roche Diagnostics). Protein concentration was determined using a BIO-RAD assay. Total protein extracts (30 µg/lane) were separated electrophoretically in 10% SDS-polyacrylamide gel and transferred onto immobilon-P membranes (Millipore). Immunodetection was performed using antibodies against pAKT (Ser473), total AKT, pRPS6 (Ser240/244), total RPS6, HK2 (Cell Signaling), CHKA (Sigma), GLUT1, LDHA (Santa Cruz Biotechnology) and GAPDH (Chemicon). Blots were revealed with peroxidase-conjugated secondary anti-rabbit or anti-mouse antibodies (Cell Signaling) followed by ECL chemiluminescence solution (Amersham Biosciences).

### *In vitro* ^1^H- and ^31^P-NMR of cell extracts

To obtain an NMR spectrum, an average of 3×10^7^ cells in logarithmic phase were extracted from cell culture using the dual phase extraction method, as previously described [30], [31]. Briefly, cells were rinsed with ice-cold saline and fixed with 10 ml of ice-cold methanol. Cells were then scraped off the surface of the culture flask and collected into tubes. Ice-cold chloroform (10 ml) was then added to each tube followed by an equal volume of ice-cold deionized water. Following phase separation, the solvent in the upper methanol/water phase was removed by lyophilization. Prior to acquisition of the NMR spectra, the water-soluble metabolites were resuspended in deuterium oxide (D_2_O) for ^1^H-NMR or D_2_O with 10 mM EDTA (pH 8.2) for ^31^P-NMR.^ 1^H-NMR and ^1^H-decoupled ^31^P-NMR spectra were acquired at 25°C on a 500 MHz Bruker spectrometer (Bruker Biospin, Coventry, UK) using a 90-degree flip angle, a 1-second relaxation delay, spectral width of 12 ppm, 64 K data points, and HDO resonance suppression by presaturation for ^1^H-NMR and a 30-degree flip angle, a 1-second relaxation delay, spectral width of 100 ppm, and 32 K data points for ^31^P. Metabolite contents were determined by integration and normalized relative to the peak integral of an internal reference [TSP (0.15%) for ^1^H-NMR, and MDPA (2 mmol/L) for ^31^P-NMR] and corrected for signal intensity saturation and the number of cells extracted per sample.

### *In vitro* Dynamic Nuclear Polarization (DNP) and Carbon (^13^C)-NMR

Real-time pyruvate-lactate exchange was measured in live cells at 37°C with a DNP-based assay as previously described [32], [33]. Live SF188 cells were studied in control or post treatment with PI-103. Cells (3.4±1×10^7^) were suspended in 500 µl of FCS free media containing DMSO (control) or PI-103 (treated) within 10 minutes of cell harvesting. [1-^13^C]pyruvic acid (99% isotopically enriched (Sigma) containing 15 mM trityl free radical OX63 (Oxford Instruments, UK) was polarized in a HyperSense® DNP polarizer (Oxford Instruments Molecular Biotools Ltd, UK) for 1 hour. The polarized sample was dissolved in 4 ml aqueous buffer (50 mM sodium lactate, 50 mM NaOH, 1 mM EDTA) resulting in a 50 mM pyruvate solution at pH 7, 37°C. A solution of 100 µl, 50 mM hyperpolarized [1-^13^C]pyruvate was mixed with 500 µl cell suspension to yield a final concentration 8 mM hyperpolarized pyruvate, 8 mM unpolarized lactate. ^13^C spectra were acquired every 2 seconds using a single scan and a 10° flip angle. Spectra were phase and baseline corrected, and peak areas integrated over the time-course of the experiment. Kinetic modeling was carried out in Matlab (Mathworks®, UK) by fitting the time-series of peak areas with the modified Bloch equations using maximum likelihood estimation. The time-courses of integrals from hyperpolarized lactate and pyruvate signals were summed and the ratios of the total lactate/total pyruvate curves were calculated to give the area under the curve metric (AUC).

### Statistical analysis

Data are presented as the mean ± SD and n ≥ 3. Statistical significance of differences was determined by unpaired two-tailed Student’s standard *t*-tests with a p-value of ≤0.05 considered to be statistically significant.

## Results

### ^1^H- and ^31^P-NMR detect metabolic changes following inhibition of the PI3K signaling pathway in pediatric glioblastoma cell lines

**Figure 1:**
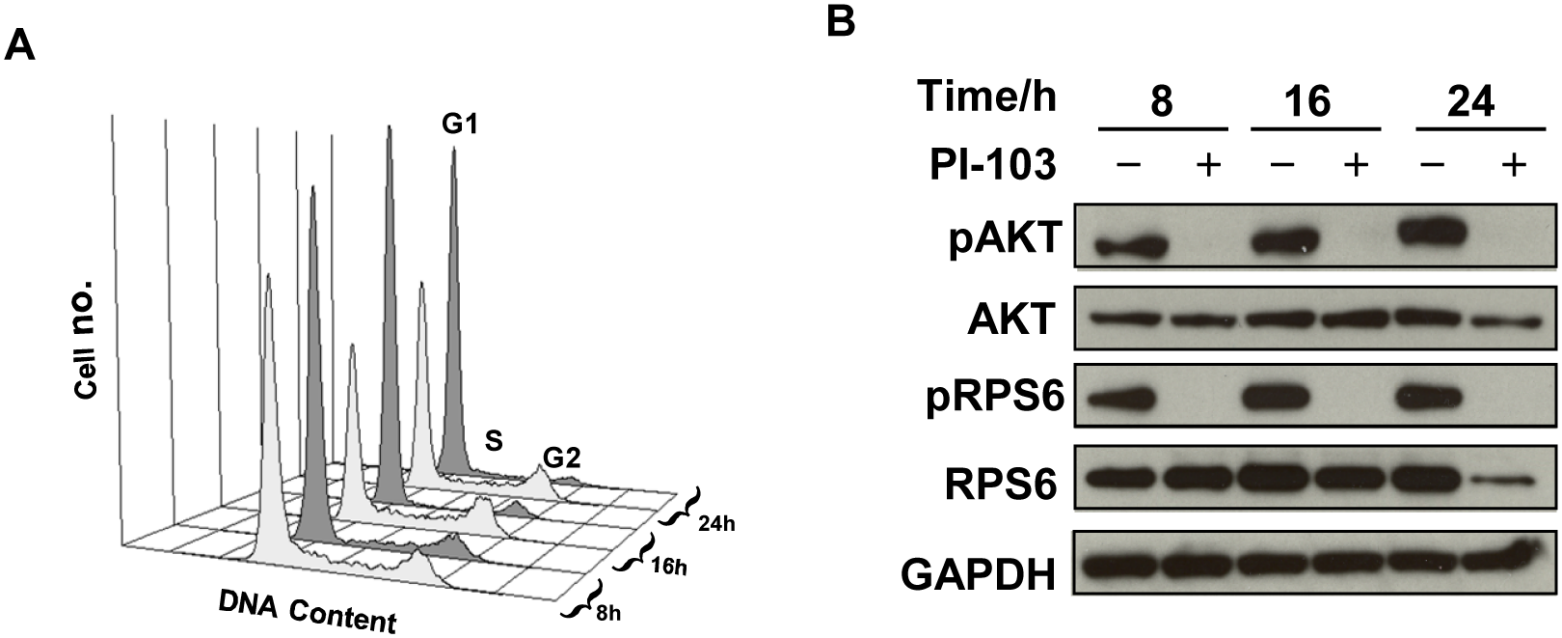
Molecular changes following treatment of SF188 pediatric glioblastoma cells with PI-103. (A) Representative flow cytometry analysis histograms showing cell cycle distribution of cells following vehicle treatment (DMSO, control, light grey), or treatment with PI-103 (5×GI_50_, dark grey) at 8, 16 or 24 hours post treatment. (B) Representative Western blots showing inhibition the PI3K signaling pathway as indicated by decreased phosphorylation of AKT (Ser473) and RPS6 (Ser240/244) at selected time points post treatment with PI-103 (5×GI_50_).

Treatment of the pediatric glioblastoma cell line SF188 with the dual pan-Class I PI3K/mTOR inhibitor PI-103 [14]–[16] for 8, 16 and 24 hours at pharmacologically active concentrations corresponding to 5×GI_50_ (GI_50_ = 0.2 µM) resulted in an increase in G1 cell population and a decrease in S phase relative to control cells (Figure 1A, Table 1). This led to a decrease in the number of treated cells per flask compared to controls (down to 82±2%, p = 0.001 and 63±4%, p = 0.0004 at 16 hours and 24 hours post treatment, respectively). Inhibition of signaling downstream of PI3K was confirmed by immunoblotting as indicated by decreased phosphorylation of AKT (Ser473) and RPS6 (Ser240/244) in treated cells compared to their controls (Figure 1B).

**Table 1:** Time-response analysis of cell cycle effects following treatment of SF188 pediatric glioblastoma cells with PI-103 (5×GI_50_).

Time/hour	8		16		24	
	C	T	C	T	C	T
**G1**	61±2	75±3*	51±4	85±3**	55±3	86±3***
**S**	29±3	17±3*	37±5	9±1**	32±1	11±4**
**G2**	10±1	8±1	12±2	6±2*	13±3	3±2*

Data are expressed as % total cell count and presented as the mean ± SD, n≥3.

Two-tailed unpaired *t* test was used to compare results in treated cells to controls.

*P<0.05,

**P<0.005,

***P<0.0005.

**Figure 2:**
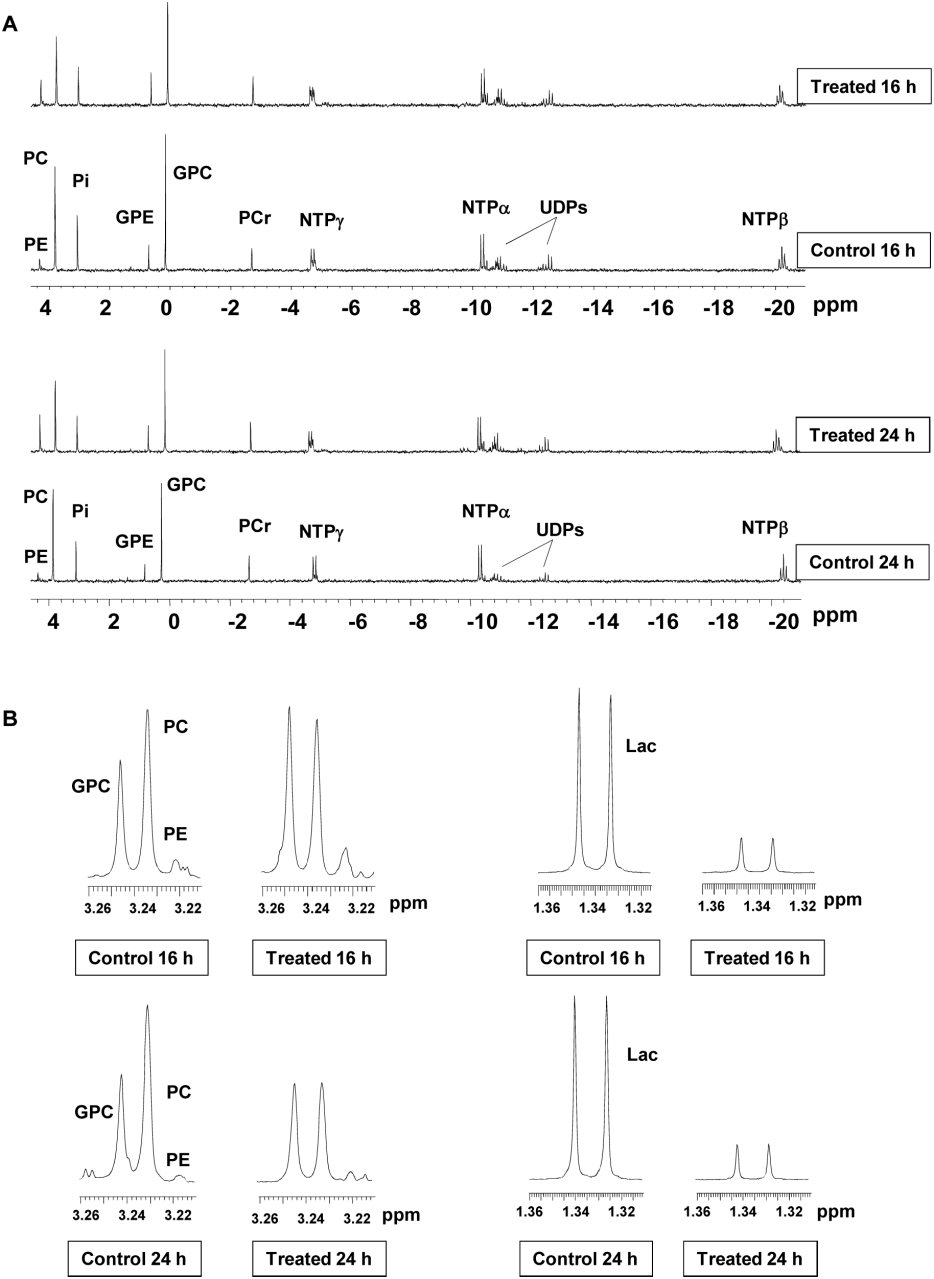
Metabolic changes following treatment of SF188 pediatric glioblastoma cells with PI-103. Representative *in vitro* (A) ^31^P-NMR spectra and (B) expansion of ^1^H-NMR spectra regions representing choline–containing metabolites (left) or lactate (Lac; right) of SF188 aqueous cell extracts following 16 or 24 hours treatment with PI-103 (5×GI_50_) compared to vehicle (DMSO) treated control. UDPs = UDP sugars.

NMR spectroscopy of aqueous extracts from cells treated *in vitro* with PI-103 was used to identify potential biomarkers of PI3K pathway inhibition. Examples of the ^31^P-NMR spectra of control and PI-103 treated SF188 cells are illustrated in Figure 2A (16 and 24 hour time points). ^31^P-NMR spectra showed that PI-103 treatment caused a decrease in PC levels compared to controls starting from 8 hours following treatment (from 24.4±5.1 fmol/cell to 13.4±2.3 fmol/cell, p = 0.03) and these were further reduced from 17.5±1.3 fmol/cell to 9.8±1.8 fmol/cell (p = 0.01) at 24 hours post treatment. A decrease in GPC was also observed (from 18.3±1.4 fmol/cell to 13.4±1.7 fmol/cell, p = 0.009) at 8 hours post PI-103 treatment while an increase in phosphoethanolamine (PE) levels was detected at 16 hours and further increased at 24 hours from 1.6±0.3 fmol/cell to 5.4±0.8 fmol/cell (p = 0.01). Time-course changes in the levels of PC and other ^31^P-NMR detected metabolites relative to their controls are summarized in Table 2.

**Table 2:** Time-response analysis showing percentage changes in ^31^P-NMR-detected metabolite levels following treatment of SF188 pediatric glioblastoma cells with PI-103 (5×GI_50_).

Time/hour	8	16	24
**PE**	90±21	183±53*	306±82*
**PC**	56±6**	70±17*	60±10*
**GPC**	74±11*	138±58	110±53
**NTP**	75±9*	106±31	94±15

Data are expressed as percentage of treated to control (% T/C) and presented as the mean ± SD, n≥3.

Glycerophosphoethanolamine (GPE) level was not affected by treatment with PI-103.

Two-tailed unpaired *t* test was used to compare results in treated cells to controls.

*P<0.05,

**P<0.005.

^1^H-NMR spectra of extracts from control and PI-103 treated SF188 cells were also investigated at several time points (Figure 2B shows the 16 & 24 hour time points). As had been seen with ^31^P-NMR, a decrease in PC levels was observed by ^1^H-NMR starting from 8 hours following treatment with PI-103 (Table 3). Furthermore, levels of tCho (PC+GPC+choline+ethanolamine metabolites) decreased at 8 hours but recovered to control levels from 16 hours post treatment (Table 3). The apparent recovery at 16 hours and later is due to the increase in PE peaks (NCH_2_, 3.22 ppm) that reside close to the tCho peaks area (N^+^(CH_3_)_3_, 3.21–3.24 ppm) within the ^1^H-NMR spectrum and is included in the summed tCho peak [20] (Figure 2B). Moreover, treatment with PI-103 caused a highly significant (p<0.004) reduction in lactate levels over the time course of treatment (Figure 2B, Table 3). No significant changes were observed in other ^1^H-NMR-detected metabolites.

**Table 3:** Time-response analysis showing percentage changes in ^1^H-NMR-detected metabolite levels following treatment of SF188 pediatric glioblastoma cells with PI-103 (5×GI_50_).

Time/hour	8	16	24
**Lac**	35±5***	39±11**	27±6**
**PC**	63±4***	69±13*	71±9*
**GPC**	88±8	125±43	105±12
**tCho**	65±18*	89±17	85±9

Data are expressed as percentage of treated to control (% T/C) and presented as the mean ± SD, n≥3.

Two-tailed unpaired *t* test was used to compare results in treated cells to controls.

*P<0.05,

**P<0.005,

***P<0.0005.

**Figure 3:**
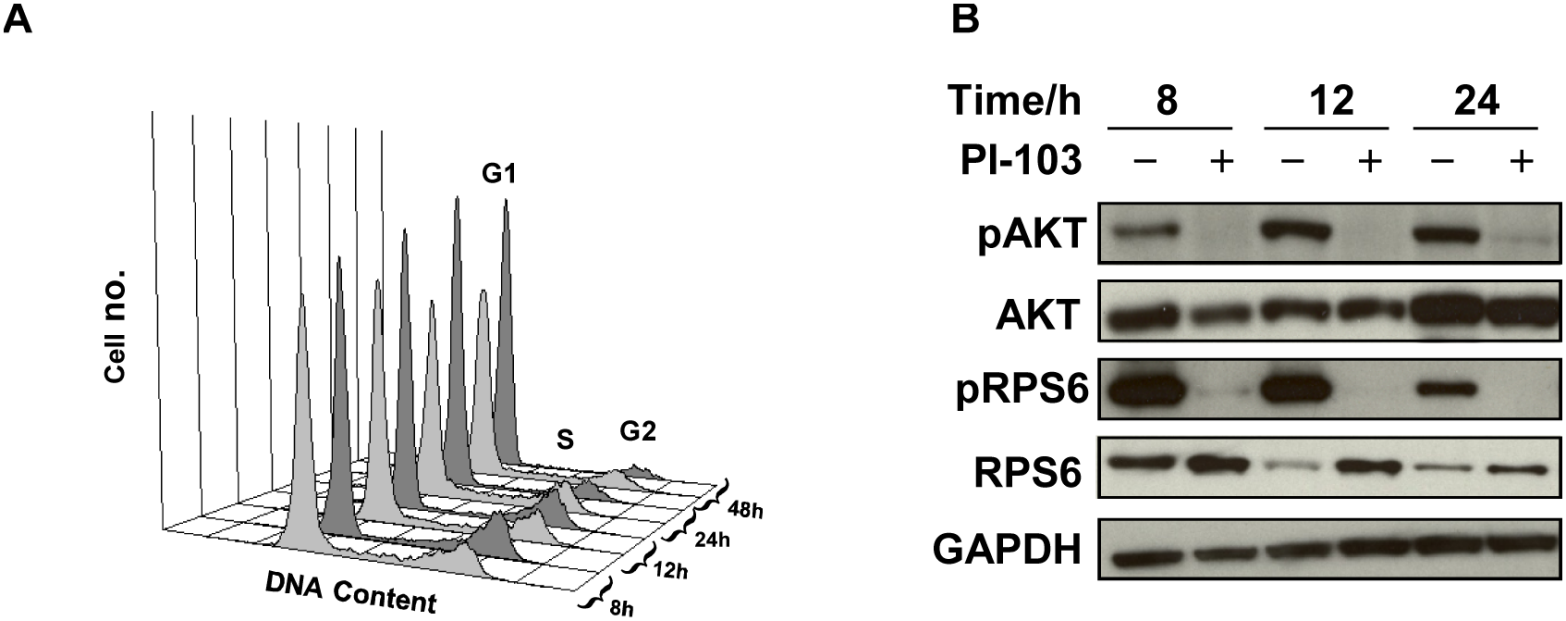
Molecular changes following treatment of KNS42 pediatric glioblastoma cells with PI-103. (A) Representative flow cytometry analysis histograms showing cell cycle distribution of cells with vehicle treatment (DMSO, control, light grey), or following treatment with PI-103 (5×GI_50_, dark grey) at 8, 12, 24 or 48 hours post treatment. (B) Representative Western blots showing inhibition of the PI3K signaling pathway as indicated by decreased phosphorylation of AKT (Ser473) and RPS6 (Ser240/244) at selected time points post treatment with PI-103 (5×GI_50_).

To test for the consistency of the NMR detected data, we also treated the pediatric glioblastoma cell line KNS42 with PI-103 for 8, 12, 24 and 48 hours at a pharmacologically active concentration corresponding to 5×GI_50_ (GI_50_ = 1.4 µM). As for SF188, PI-103 caused a G1 arrest in KNS42 cells (Figure 3A, Table 4), resulting in a decrease in the proliferation of treated cells compared to controls (down to 49±8%, p = 0.002, 48 hours). Inhibition of PI3K signaling was confirmed by decreased phosphorylation of AKT (Ser473) and RPS6 (Ser240/244) in treated cells compared to their controls (Figure 3B).

**Table 4:** Time-response analysis of cell cycle effects following treatment of KNS42 pediatric glioblastoma cells with PI-103 (5×GI_50_).

Time/hour	8		12		24		48	
	C	T	C	T	C	T	C	T
**G1**	60±5	63±4	62±2	70±4*	60±4	84±1*	69±3	89±2***
**S**	30±4	26±4	26±2	18±3**	27±8	10±3	22±1	5±2***
**G2**	10±3	11±1	12±3	12±2	13±4	6±2	9±3	6±2

Data are expressed as % total cell count and presented as the mean ± SD, n≥3.

Two-tailed unpaired *t* test was used to compare results in treated cells to controls.

*P<0.05,

**P<0.005,

***P<0.0005.

**Figure 4:**
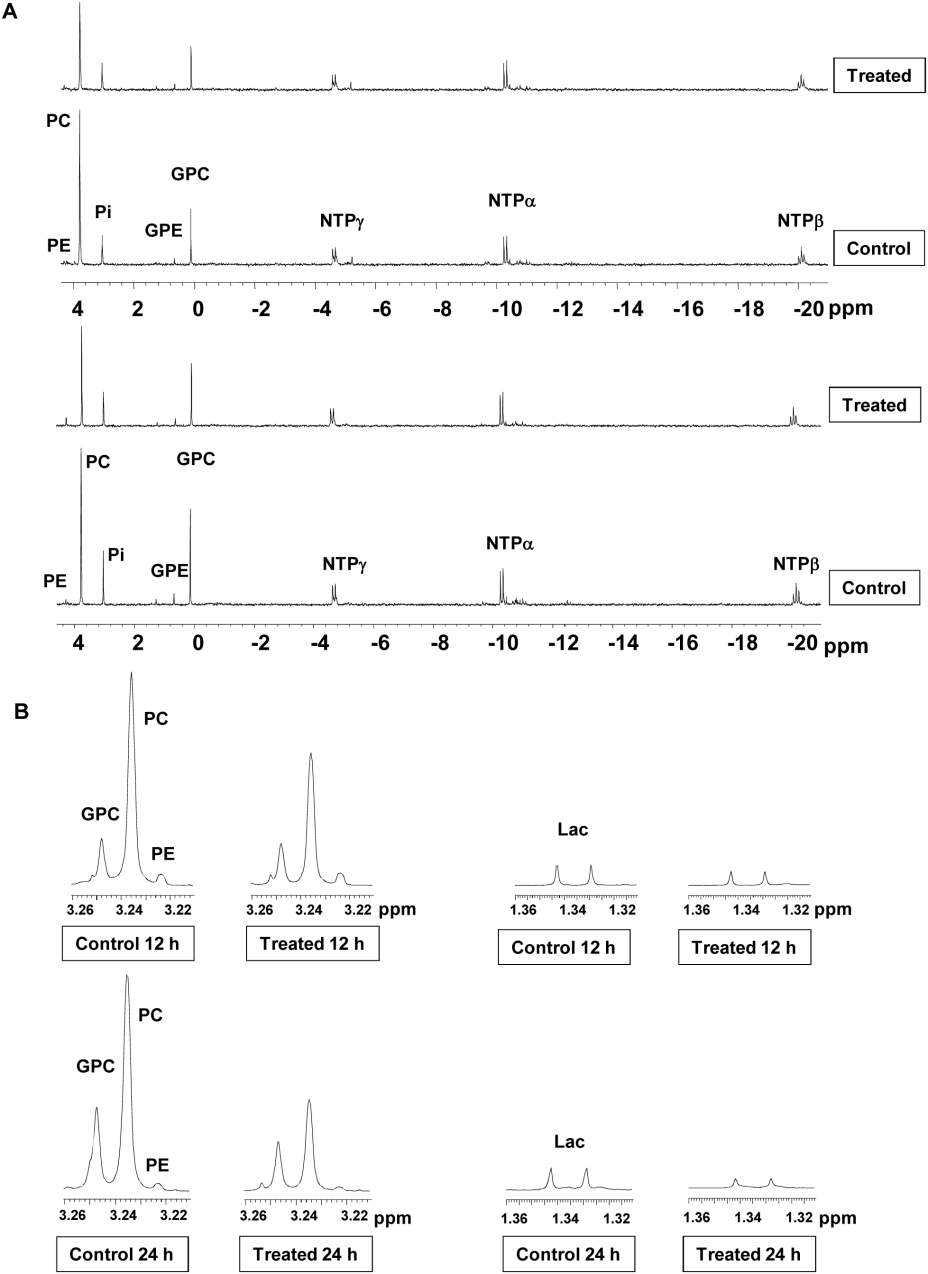
Metabolic changes following treatment of KNS42 pediatric glioblastoma cells with PI-103. Representative *in vitro* (A) ^31^P-NMR spectra and (B) expansion of ^1^H-NMR spectra regions representing choline–containing metabolites (left) or lactate (Lac; right) of KNS42 aqueous cell extracts following 12 or 24 hours treatment with PI-103 (5×GI_50_) compared to vehicle (DMSO) treated control.

Analysis of ^31^P-NMR spectra of control and PI-103 treated KNS42 cells showed a decrease in PC levels relative to controls that was similar to that observed with SF188, starting from 8 hours but in this case reaching significance at 12 hours following treatment from 40.2±2.0 fmol/cell to 26.3±7.9 fmol/cell (p = 0.02) and was down from 37.8±5.9 fmol/cell to 20.5±6.1 fmol/cell (p = 0.02) following 48 hours of incubation with PI-103 (Figure 4A shows the 12 & 24 hour time points). Time-course changes in the levels of PC and other ^31^P-NMR detected metabolites relative to their controls are summarized in Table 5. ^1^H-NMR confirmed changes detected by ^31^P-NMR and further showed a significant (p<0.01) decrease in levels of tCho and lactate (Figure 4B, Table 6).

**Table 5:** Time-response analysis showing percentage changes in ^31^P-NMR-detected metabolite levels following treatment of KNS42 pediatric glioblastoma cells with PI-103 (5×GI_50_).

Time/hour	8	12	24	48
**PC**	76±32	65±10*	50±11*	56±5*
**GPC**	84±32	78±22	69±12	168±70
**NTP**	86±28	87±35	81±9	111±31

Data are expressed as percentage of treated to control (% T/C) and presented as the mean ± SD, n≥3.

PE in KNS42 cells was not consistently detectable due to the low levels of this metabolite and was not affected by treatment with PI-103.

Glycerophosphoethanolamine (GPE) level was not affected by treatment with PI-103.

Two-tailed unpaired *t* test was used to compare results in treated cells to controls.

*P<0.05.

**Table 6:** Time-response analysis showing percentage changes in ^1^H-NMR-detected metabolite levels following treatment of KNS42 pediatric glioblastoma cells with PI-103 (5×GI_50_).

Time/hour	8	12	24	48
**Lac**	73±32	70±10*	42±11*	36±5***
**PC**	77±20	62±10**	49±6*	50±8**
**GPC**	84±22	75±9*	62±6*	133±25
**tCho**	81±21	65±10**	54±9*	64±10*

Data are expressed as percentage of treated to control (% T/C) and presented as the mean ± SD, n≥3.

Two-tailed unpaired *t* test was used to compare results in treated cells to controls.

*P<0.05,

**P<0.005,

***P<0.0005.

### Treatment of the pediatric glioblastoma cell lines with the pan-Class I PI3K inhibitor GDC-0941 results in similar metabolic changes compared to the dual pan-Class I PI3K/mTOR inhibitor PI-103

**Figure 5:**
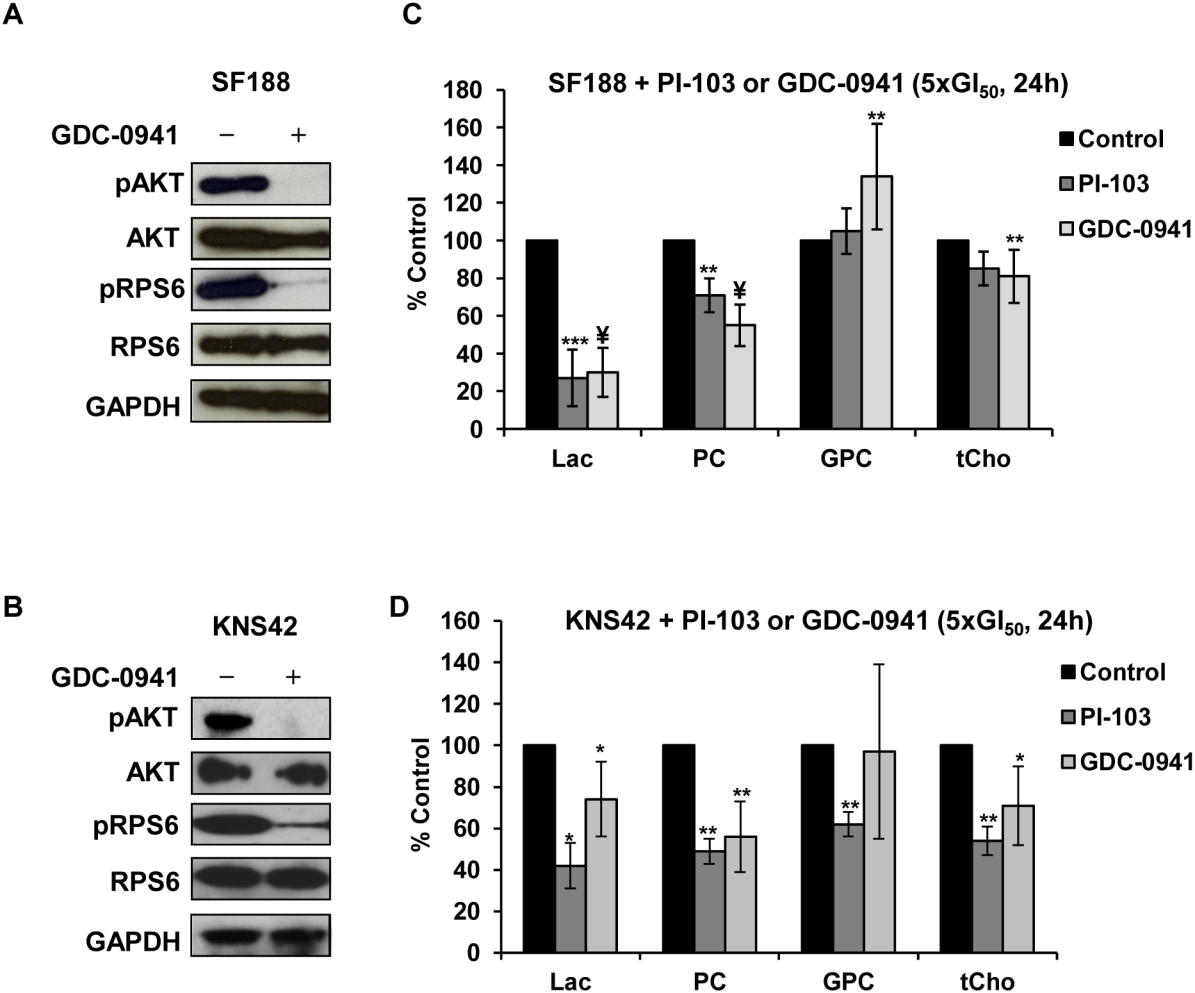
Molecular and metabolic percentage changes caused by treatment of pediatric glioblastoma cells with GDC-0941 or PI-103 (5×GI_50_, 24 hours). Representative Western blots showing inhibition of the PI3K signaling pathway as indicated by decreased phosphorylation of AKT (Ser473) and RPS6 (Ser240/244) post-treatment with GDC-0941 in: (A) SF188 or (B) KNS42 cells. Comparison of ^1^H-NMR detected metabolite percentage changes caused by treatment with PI-103 or GDC-0941 in: (C) SF188 or (D) KNS42 cells. Results are expressed as percentage of treated to control and presented as the mean ± SD (error bars) of at least three separate experiments. Statistically significantly different from the control *p≤0.05, **p≤0.01, ***p<0.005, ^¥^p<0.0005; two-tailed unpaired *t* test was used for all comparisons.

We see consistent and significant changes in PC, tCho and lactate in two different pediatric cell lines. Hence we tried a different PI3K inhibitor to assess the potential relevance of the NMR-detectable metabolic changes to inhibition of the PI3K signaling pathway. Both pediatric glioblastoma cell lines were treated with the selective pan-Class I PI3K inhibitor GDC-0941 [14]–[16] which does not significantly inhibit mTOR and is entering Phase II clinical trials. Treatment with GDC-0941 at 5×GI_50_ for 24 hours (SF188 GI_50_ = 1.2 µM, KNS42 GI_50_ = 1.8 µM), decreased cell number to 60±10% (p = 0.00003) and 81±11% (p = 0.03) in SF188 and KNS42, respectively. Inhibition of PI3K signaling was confirmed by decreased phosphorylation of AKT (Ser473) and RPS6 (Ser240/244) in treated cells compared to their controls (Figure 5A & B).

NMR analysis showed decreases in PC, tCho and lactate levels in both cell lines, similar to that observed following PI-103 treatment (Figure 5C & D). Again like PI-103, treatment with GDC-0941 caused a significant increase in PE levels in SF188 cells (up to 145±27%, p = 0.03) but not with KNS42 cells (124±48%, p = 0.4). However, the effects of GDC-0941 on GPC levels were different to that of PI-103, with an increase (up to 133±23%, p = 0.05) in SF188 cells and no change (119±37%, p = 0.4) in KNS42 cells.

### Treatment of SF188 pediatric glioblastoma cells with the cytotoxic drug TMZ results in metabolic changes distinct from those seen with the PI3K pathway inhibitors PI-103 and GDC-0941

**Figure 6:**
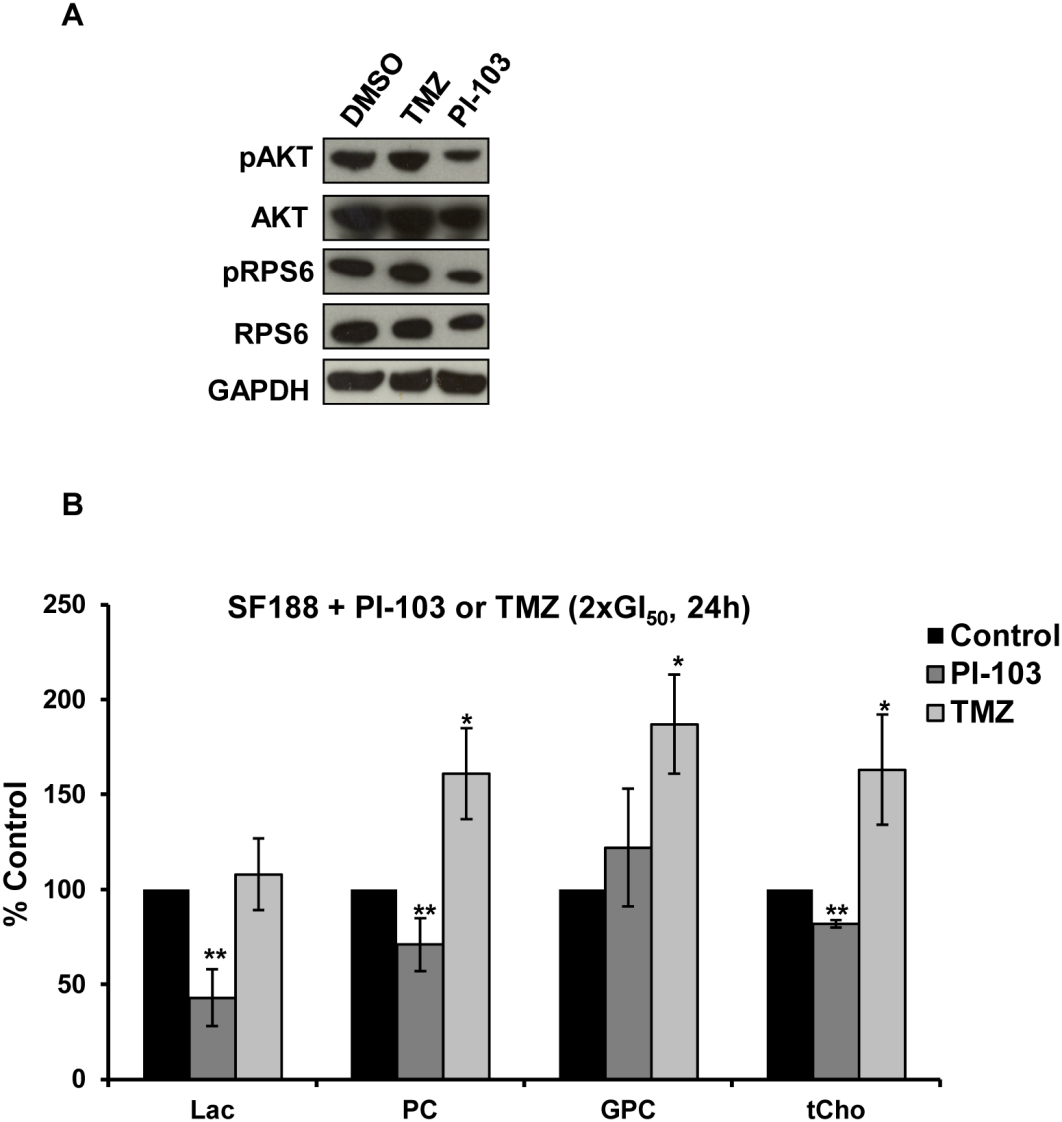
Comparison of molecular and metabolic changes caused by 24 hours treatment of SF188 pediatric glioblastoma cells with PI-103 or TMZ at 2×GI_50_. (A) Representative Western blots showing inhibition of the PI3K signaling pathway as indicated by decreased phosphorylation of AKT (Ser473) and RPS6 (Ser240/244) following treatment with PI-103 but not with TMZ. (B) Quantification of ^1^H-NMR detected metabolite changes. Results are expressed as percentage of treated to control and presented as the mean ± SD (error bars) of at least three separate experiments. Statistically significantly different from the control *p≤0.05, **p≤0.01, ***p<0.005, ^¥^p<0.0005; two-tailed unpaired *t* test was used for all comparisons.

To further assess specificity of our NMR-detected metabolic changes to PI3K inhibition rather than anti-proliferative effects, the SF188 pediatric glioblastoma cell line was treated with PI-103 or the cytotoxic drug TMZ. TMZ was selected as this is the standard frontline treatment for patients with glioblastoma [10], [11]. Concentrations equivalent to 2×GI_50_ were selected for this comparison in order to be more representative of clinical conditions and to assess whether the metabolic changes could be detected even at lower drug concentrations than previously tested. Treatment of SF188 cells with PI-103 at 2×GI_50_ for 24 hours decreased cell number to 58±8%, p = 0.00007 relative to controls and inhibited PI3K signaling (Figure 6A). Our NMR-detected changes were still observed when PI-103 concentration was reduced to 2×GI_50_ (Figure 6B) compared to 5×GI_50_ (Figure 5C). Treatment of SF188 cells with TMZ at 2×GI_50_ for 24 hours (GI_50_ = 0.46 mM) decreased cell number to 66±17%, p = 0.008 and as expected, TMZ did not affect PI3K signaling (Figure 6A). In direct contrast to the decrease in PC and tCho consistently observed with PI-103 and GDC-0941, ^1^H-NMR spectra showed that TMZ significantly (p≤0.04) increased levels of PC, GPC and tCho. TMZ had no effect on lactate levels (Figure 6B).

### Inhibition of the PI3K signaling pathway in SF188 pediatric glioblastoma cells results in altered expression of enzymes involved in choline and glucose metabolism

**Figure 7:**
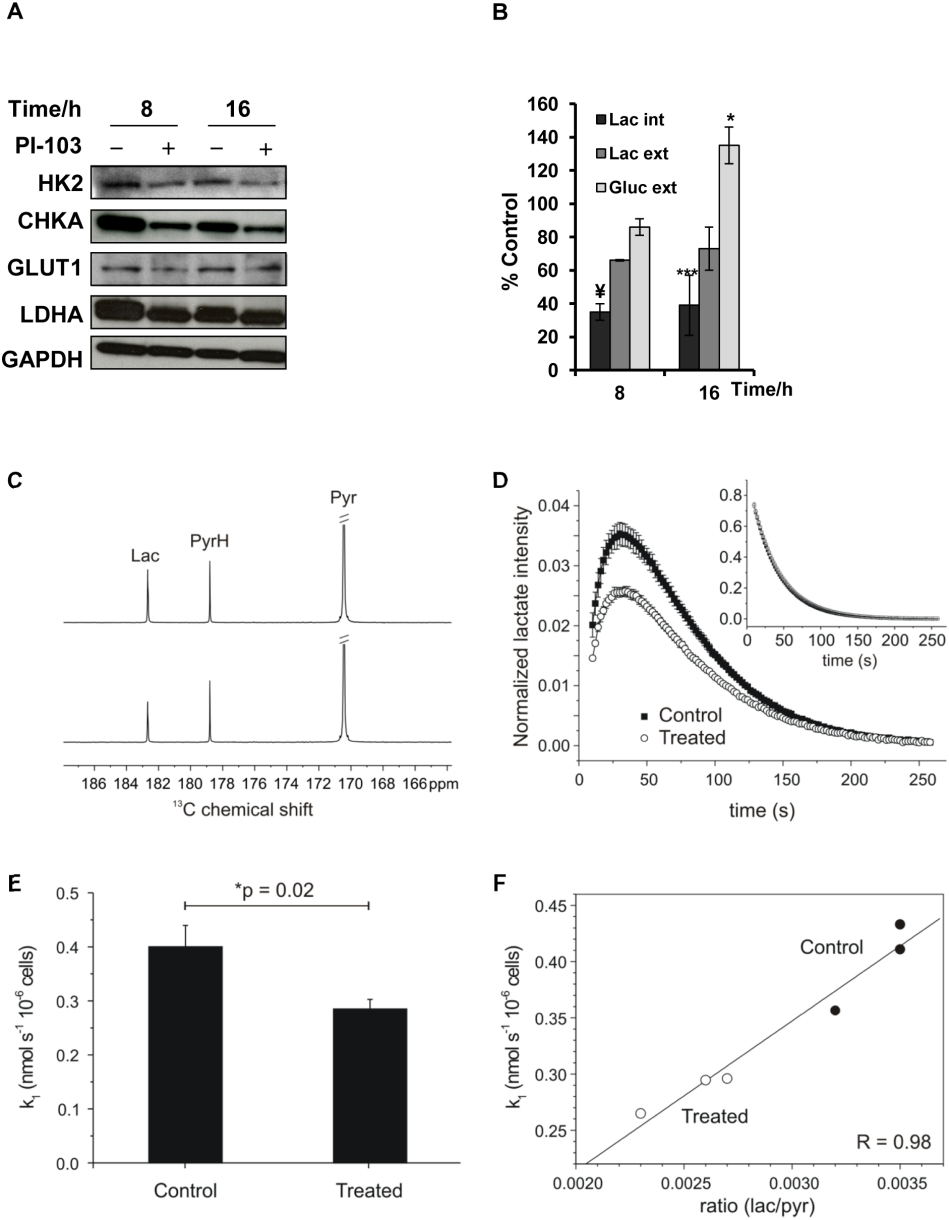
Investigation of mechanisms underlying NMR-detected changes in the levels of choline metabolites and lactate following treatment of SF188 pediatric glioblastoma cells with PI-103 (5×GI_50_). (A) Representative Western blots showing changes in protein expression levels of enzymes involved in choline metabolism (CHKA) and glucose metabolism including: GLUT1, HK2 and LDHA, at selected time points post treatment with PI-103. GAPDH was used as a loading control. (B) Quantitative measurement of ^1^H-NMR detected percentage changes in the levels of lactate (Lac, internal & external) and glucose (external) at selected time points post treatment with PI-103 relative to controls, 8 hours n = 2. Results are expressed as percentage of treated to control and presented as the mean ± SD (error bars). Statistically significant different from the control *p≤0.05, **p<0.01, †p≤0.005, ‡p<0.0005; two-tailed unpaired *t* test was used for all comparisons. (C) Sum ^13^C-NMR spectra over the entire time-series with peaks corresponding to lactate, pyruvate and pyruvate hydrate in either control (top) or PI-103 treated groups (bottom), from which a decrease in the lactate peak intensity can be appreciated. (D) The time-series of the lactate peak integral normalized to cell number with inset showing the pyruvate time dependence. (E) A decrease in the rate of pyruvate-lactate exchange rate constants was detected in PI-103 treated cells compared to controls. (F) A decrease in the area under the curve was also observed in PI-103 treated cells compared to controls, p = 0.005.

We used different techniques to explore the mechanisms underlying the metabolic changes observed with NMR. The SF188 cell line treated with PI-103 (5×GI_50_) was selected as a model. Previously [24], we have shown a direct correlation between changes in PC levels detected with NMR and changes in protein expression levels of CHKA, the enzyme responsible for phosphorylating choline and generating PC. To find out whether this applies to the pediatric cell line SF188, CHKA protein levels were analyzed by immunoblotting. A decrease in CHKA protein expression was detectable from 8 hours following treatment of SF188 cells with PI-103, and was maintained over the time course of treatment (Figure 7A).

Immunoblotting was also used to assess mechanisms underlying the decrease in lactate following PI3K pathway inhibition; protein expression levels of the facilitative glucose transporter GLUT1 and the glycolytic enzymes HK2 and LDHA were reduced over the time course of treatment with PI-103 (Figure 7A). Interestingly, protein expression levels of the glycolytic enzyme GAPDH was not affected by PI-103 treatment allowing its use as a loading control.

To determine whether the decrease in intracellular lactate could be due to the efflux of lactate into the tissue culture medium, we used ^1^H-NMR to measure levels of lactate in the growth media of control and PI-103 treated SF188 cells. Similar amounts of lactate were detected in the medium of treated compared to control cells. However, analysis of glucose showed higher glucose levels in media from the 16 and 24 hours PI-103 treated cells relative to controls (Figure 7B), indicating a decrease in glucose uptake by the PI-103 treated cells.

### Hyperpolarized pyruvate-lactate ^13^C exchange assay

Finally, we used the DNP technique to measure the exchange kinetics of hyperpolarized lactate from hyperpolarized pyruvate in PI-103 treated compared to control live SF188 cells. Sum ^13^C-NMR spectra over the entire time-series are displayed in Figure 7C with peaks corresponding to lactate, pyruvate and pyruvate hydrate for the SF188 cell line in either control (top) or PI-103 treated groups (bottom), from which a decrease in the lactate peak intensity can be appreciated. The time-series of the lactate peak integral normalized to cell number is shown pre and post treatment in Figure 7D with inset showing the pyruvate time dependence. Rate constants for the forwards exchange reaction from pyruvate to lactate were derived from non-linear least squares fitting of the bi-exponential time dependence of the hyperpolarized lactate and pyruvate curves and normalized to cell number in both control and treated cells [32], [33].

A decrease in the rate of pyruvate-lactate exchange rate constants (Figure 7E) was detected in PI-103 treated cells (0.285±0.018 nmol s^−1^ 10^−6^ cells) compared to controls (0.400±0.039 nmol s^−1^ 10^−6^ cells), p = 0.02. A decrease in the area under the curve was also observed (Figure 7F) in PI-103 treated cells (3.84±0.44×10^−9^ cells) compared to controls (5.83±0.25×10^−9^ cells), p = 0.005.

## Discussion

There is substantial evidence to suggest that activation of the PI3K signaling pathway is of major importance in pediatric glioblastoma [12], [13]. Numerous small-molecule inhibitors of the PI3K signaling pathway have been developed [14]–[16], and early phase clinical studies of these inhibitors are planned for children with glioblastoma. Identification of non-invasive biomarkers of target and pathway inhibition and potentially of tumor response to this novel treatment would be of great value in the clinical development of PI3K inhibitors. The challenges of obtaining pharmacodynamic biomarker information is especially challenging in childhood brain tumors, where repeated biopsy is typically too invasive and therefore not routinely carried out. Other potential functional imaging techniques such as [18] FDG-PET have the disadvantage of requiring exposure to ionizing radiation, preferably avoided in pediatric patients where possible. In this study, we used NMR spectroscopy to search for potential non-invasive biomarkers for the effects of the dual pan-Class I PI3K/mTOR inhibitor PI-103 [14]–[16] and the pan–Class I PI3K inhibitor GDC-0941 lacking significant mTOR activity [14]–[16] in two pediatric glioblastoma cell lines, SF188 and KNS42, *in vitro*. This was with a view to informing future *in vivo* validation of such biomarkers.

Using ^1^H- and ^31^P-NMR, a significant decrease in PC concentrations was detected in both cell lines following treatment with either PI-103 or GDC-0941. Furthermore, both treatments resulted in a significant increase in PE levels in SF188 cells but not in KNS42 cells. Treatment with GDC-0941 also caused an increase in GPC in SF188 but not in KNS42 cells. This and our previously published results, using different adult cancer models [24], [25], indicate that the decrease in PC levels is likely to be related to the action of LY294002, wortmannin, PI-103 and GDC-0941 on their common target PI3K. In contrast, the increase in PE or GPC was less consistent over a range of PI3K pathway inhibitors and was cell line dependent and, moreover, was seen only after longer inhibition periods (≥16 hours) and when higher concentrations of PI3K pathway inhibitors were used (5×GI_50_). This may be related to specific functions of these metabolites in those particular cell lines or to non-specific effects triggered by prolonged PI3K pathway inhibition. Interestingly, despite the difference in response of some choline- and ethanol-containing metabolites, the sum of these metabolites represented by the tCho peak in the ^1^H-NMR spectra generally decreased following treatment with both PI-103 and GDC-0941 in both pediatric glioblastoma cell lines. This indicates that the effect of both PI3K pathway inhibitors may be monitored *in vivo* or clinically in future, using either ^31^P-MRS by following changes in PC levels or the composite tCho peak with ^1^H-MRS. Clinically, we and others have shown that these metabolites can be measured non-invasively in spectra from a range of tumors in adult and pediatric subjects [22], [34]–[36].

In direct contrast to the decrease in PC and tCho levels observed with PI3K pathway inhibitors, treatment of the pediatric glioblastoma cell line SF188 with the standard-of-care DNA damaging agent TMZ resulted in an increase in PC, GPC and tCho levels. This is in line with the increase in GPC levels we previously reported following treatment with various cytotoxic anti-cancer drugs [30], [37]. Together, these results are consistent with the NMR-detected decreases in PC and tCho being a consequence of the inhibition of PI3K pathway signaling and not due to anti-proliferative effects associated with cytotoxicity.

Further inspection of ^1^H-NMR spectra showed that treatment of pediatric glioblastoma cells with the PI3K pathway inhibitors PI-103 and GDC-0941 was associated with a marked decrease (>60%) in lactate levels. In contrast, treatment with TMZ had no effects on lactate levels. These results are in line with recent findings [38]–[40] showing a decrease in lactate levels following treatment with various inhibitors of the PI3K/AKT/mTOR signaling pathway *in vitro* and *in vivo* using different tumor models including adult glioblastoma.

It is well established that cancer cells reprogram their metabolism to facilitate growth and survival, leading to alterations in glucose, glutamine and lipid metabolism [41], [42]. These metabolic pathways are regulated by signaling pathways known to be activated in and contributing to cancer development. For example, the PI3K/AKT/mTOR signaling pathway is a master regulator of enzymes involved in glucose, glutamine and lipid metabolism [43], [44]. Therefore, it is not surprising that inhibition of the PI3K signaling pathway would impact on levels and/or activities of these enzymes. In the present study, we have explored possible mechanisms underlying NMR-detectable metabolic changes with the focus on SF188 cell line treated with PI-103 as a model. We have shown that the decrease in PC following treatment with PI-103 is associated with a reduction in the protein expression level of CHKA, the enzyme responsible for phosphorylation of choline into PC, confirming our previous findings in other cancer models [24]. We have also shown that the decrease in lactate levels was associated with a decrease in the protein expression levels of the glycolytic enzymes HK2 and LDHA. Furthermore, we demonstrated that the ^1^H-NMR-detected higher levels of glucose in the medium of treated cells compared to their controls, is consistent with the decrease in the protein levels of the facilitated glucose transporter GLUT1. A previous report demonstrated that the reduction in LDH expression is mediated by a reduction in HIF1A following inhibition of the PI3K signaling pathway in adult glioblastoma [40]. The possible involvement of HIF1A in the decrease of the expression of several glycolytic enzymes detected in our model, need to be confirmed. Interestingly, the protein expression levels of the glycolytic enzyme GAPDH which we used as a loading control, were not affected by PI3K inhibition, indicating that it is not a general effect on glycolytic enzymes.

Taken together, our findings suggest that PI-103 inhibits glucose uptake and reduces levels of glycolytic enzymes resulting in a decrease in the production of lactate. This supports recent reports [45], [46] suggesting the utility [^18^F]FDG as a tracer for measuring response to PI3K inhibition. Interestingly, decreased [^18^F]FDG uptake (25%) was observed in 53% of patients following treatment with the PI3K inhibitor BKM120, and it was suggested that this effect may be due to a combination of antitumor activity and direct PI3K inhibition [14], [18]. Furthermore, our data are consistent with our previously published genome-wide cDNA microarray profiling, showing altered expression of genes involved in glucose and cholesterol biosynthesis in response to PI-103 in the PTEN null human glioblastoma cells U87MG (ref. [47] and Supplementary data published therein).

The DNP technique has recently been implemented to monitor the exchange of hyperpolarized ^13^C from pyruvate to lactate [32], [40], [48]. This novel method provides an enhancement in signal-to-noise ratio of over 10,000-fold when compared with traditional ^13^C-NMR [49], and has undergone an initial clinical trial [50]. Using DNP, we have demonstrated here a reduction in the rate of hyperpolarized pyruvate to lactate exchange kinetics in PI-103 treated SF188 cells when compared to controls. This is consistent with the decrease in LDHA protein expression levels in PI-103 treated cells. Our findings are in line with a previous report showing a decrease in ^13^C lactate exchange and LDH expression and activity following PI3K/AKT/mTOR inhibition in adult glioblastoma [40]. This suggests that this novel method could be used to monitor modulation of the PI3K/mTOR pathway in pediatric glioblastoma. However, the mechanisms of drug action leading to changes in pyruvate-lactate exchange are complicated and have been shown to be influenced not only by LDH activity, but also NAD/NADH ratio, endogenous concentrations of lactate as well as the influence of monocarboxylate transporters MCT1 and 4.

As discussed in our previous publications [24], [51], although our NMR-detected metabolic changes may not be specific to the PI3K pathway, they can provide valuable biomarkers of response [17]. Many biomarkers are not specific to a given pathway, nonetheless have considerable clinical value (e.g. [^18^F]FDG).

*In vitro* research represents the first approach to testing a drug effect on target pathways, providing a well-defined environment and minimizing the use of animals. The important *in vivo* validation of the potential non-invasive metabolic biomarkers we have identified *in vitro* requires a PI3K inhibitor suitable for clinical studies that, in relation to glioblastoma, would ideally also cross the blood brain barrier. Despite being highly potent and selective inhibitors of PI3K signaling, neither PI-103 nor GDC-0941 fulfill these criteria as PI-103 does not have optimal drug-like properties and is not a clinical candidate, and GDC-0941 does not cross the blood-brain barrier. Having identified potential biomarkers of PI3K inhibition in pediatric glioblastoma cell lines, further validation will be best performed in an orthotopic model using an inhibitor that is a clinical candidate and crosses the blood-brain barrier. Orthotopic models are in the late stages of development in our Centre and will be invaluable for future *in vivo* research.

In conclusion, we have shown that PI3K/mTOR inhibition in pediatric glioblastoma cell lines interferes with glucose and choline metabolism leading to decreases in lactate and choline metabolite levels that are detected by NMR. Alterations in these metabolites may have considerable potential as non-invasive biomarkers for monitoring response to PI3K/mTOR inhibitors in early phase clinical trials in children with glioma, thereby helping to optimize dosing and treatment of this disease which has devastatingly poor prognosis.
